# Intragastric rupture of splenic artery aneurysms: Three case reports and literature review

**Published:** 2013-04

**Authors:** Yang-de MIAO, Bei YE

**Affiliations:** 1 Yang-de MIAO, Department of Gastroenterology, Taizhou Municipal Hospital, Taizhou 318000, Zhejiang, China.; 2 Bei YE, Department of Gastroenterology, Taizhou Municipal Hospital, Taizhou 318000, Zhejiang, China.

**Keywords:** Splenic arterty aneurysm, Gastrointestinal bleeding, Computed tomography

## Abstract

Rupture of splenic artery aneurysm remains an uncommon cause of hypovolemic shock although it is the third most common intra-abdominal aneurysms. It is difficult to diagnosis timely and entails a significant morbidity and mortality. We present three uncommon cases of bleeding from upper gastrointestinal tract as a result of rupture of splenic artery aneurysm to stomach in patients with liver cirrhosis or infectious endocarditis. We also reviewed the literature and these case reports highlighted that rapid resuscitation, diagnostic imaging, surgical consultation, and alternatively transarterial embolization were the priorities in the management. Early diagnosis and intervention for ruptured splenic artery aneurysm are crucial for patient’s survival; therefore, it must be kept in mind as feasible etiology of life-threatening gastrointestinal bleeding, especially in patients with underlying liver cirrhosis or infective endocarditis.

## INTRODUCTION

Splenic artery aneurysms (SAAs) are extremely rare disease that is usually asymptomatic and can be fatal because of hemorrhagic shock once it ruptures. Although the chance of diagnosis of SAAs before rupture has greatly increased with the recent advances in diagnostic imaging, it is still a very uncommon entity in the differential diagnosis of upper gastrointestinal bleeding because of its very low prevalence and emergency physicians would be too easily to attribute the bleeding in patients with overt portal hypertension (PHTN) and varices to the coagulation dysfunction and possible rupture of varices. Therefore, physicians usually have insufficient awareness of this rare disorder. Herein, we present three cases of uncommon massive upper gastrointestinal hemorrhage due to rupture of SAAs admitted to Taizhou Municipal Hospital and Sir Run Run Shaw hospital between November 2004 and July 2011. We also reviewed the literature with a comprehensive overview on major clinical characteristics and current management options, in order to improve the diagnostic accuracy and management efficiency for those patients.

## CASE REPORT

**Fig.1:**
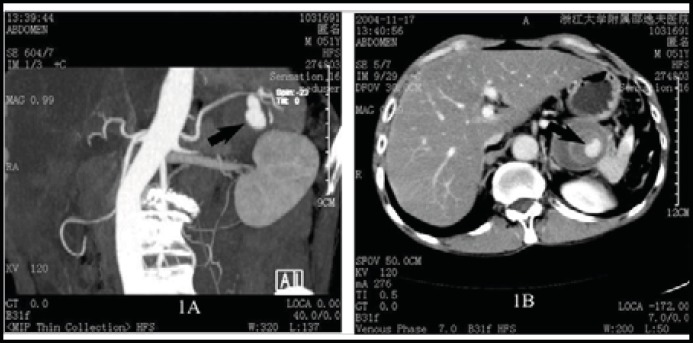
Contrast enhanced CT shows splenic artery aneurysm located near apex of left kidney (black arrow, 1A) and formation of hematoma next to the posterior wall of stomach (black arrow, 1B

***Case 1: ***A 51-year-old cirrhotic man presented with abdominal pain for one day. He then had a sudden upper gastrointestinal bleeding and a contrast-enhanced computed tomography (CT) demonstrated that a ruptured SAA located near to splenic hilum and formation of hematoma in left epigastric region (Fig.1). Further ultrasonography confirmed liver cirrhosis and ascites. He underwent an urgent laparotomy. After dissection of severely adhered tissues, a pulsatile mass approximate 4.8 cm×5.6 cm×4.9 cm was found closely adhensive to stomach and splenic hilum. SAA resection, splenectomy, gastric suture and conventional splenorenal shunt were performed thereafter and the recovery was uneventful.

***Case 2: ***A 42-year-old man presented with sudden abdominal pain and hematochezia for one day. He had a past history of splenoectomy three years prior for traffic injury. He was alcoholic and was once diagnosed as alcoholic cirrhosis. On admission, he was hypotensive at 91/60 mm Hg, and tachycardia with a pulse of 118 beats/min. Upon physical examination, he was found to have a distended abdomen and slight tenderness in periumbilical region, and the shifting dullness sign were noted. Ultrasonography of the abdomen revealed liver cirrhosis, massive ascite. Apart from slightly decreased hemoglobin 11.6g/dL, all other laboratory examinations were unremarkable. He was admitted to gastroenterology ward under the diagnosis of bleeding esophageal varices or peptic ulcer and decompensated alcoholic cirrhosis with ascites.

**Fig.2:**
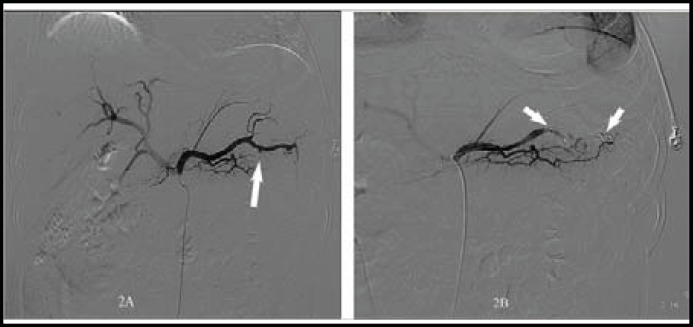
Angiography demonstrated a 2-cm-diameter SAA arising from the middle-distal splenic artery (arrow, 2A). The aneurysm disappeared after embolization with multiple coils (arrow, 2B) placed in splenic artery segments proximal and distal to the aneurysmal neck

After his admission, he complained of a progressive distending abdomen and underwent a paracentesis which showed a bloody ascite. Another check of hemoglobin decreased to 8.9 g/dL and an urgent contrast-enhanced CT demonstrated giant hematoma in lesser sac and peri-pancreatic space. Subsequently, he underwent emergency laparotomy and two liters of blood and was found in the abdomen. Severe adhesions were encountered in the peritoneal cavity. Following a careful dissection of severely adhered tissues and removal of clots, blood was suspected to be oozing from the gastric coronary vein, which was sutured subsequently and the patient was sent to intensive care unit. From then on, hemoglobin fluctuated around 11.5 g/dL in the following 66 hrs, followed by a sudden decrease to 9.6 g/dL and 7.5 g/dL on 4^th^ day postoperation. Bedside ultrasonography and contrast-enhance CT demonstrated hematoma again in lesser sac and peri-pancreatic space. Therefore, digital subtraction angiography (DSA) of celiac and superior mesenteric arteries were performed which showed a 2-cm-diameter saccular aneurysm that arised from the middle-distal splenic artery. The aneurysm disappeared after embolization with multiple coils placed in splenic artery segments proximal and distal to the aneurysmal neck (Fig.2). Unfortunately, the patient underwent a second laparotomy because of his hemoglobin kept dropping. After careful clean of 1000 ml blood and clots, a ruptured and bleeding SAA was found to be tightly adhensive to the neighbouring posterior wall of the stomach. Partial pancreatectomy involving pancreatic tail and aneurysm resection, and gastric suture were performed, after which the patient had an unremarkable recovery and was discharged 20 days later.

**Fig.3:**
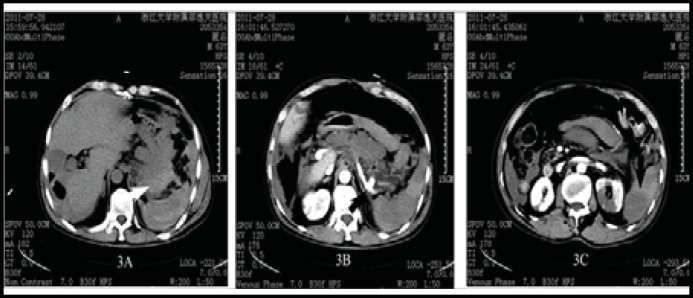
CT demonstrated intraabdominal bleeding in the lesser omental sac (3A), splenic artery aneurysm (black arrow, 3B), and infarction of spleen and left kidney (black arrows, 3C).

***Case 3: ***A 63-year-old man presented to the emergency department with epigastric pain, malaise, and hematochezia for one day. Except for a history of aortic valve replacement three years ago, there was no other remarkable pertinent finding in his medical history. The patient was afebrile, conscious and haemodynamically stable at triage. After a non-enhanced abdominal CT which demonstrated left renal infarction and suspected acute pancreatitis, the patient was admitted to gastroenterology ward with the diagnosis of upper gastrointestinal bleeding, left renal infarction and possible acute pancreatitis. Upon his arrival on the ward, he suddenly became hypotensive and collapsed, and was found to have profuse sweating. After fluid resuscitation, the patients regained consciousness soon. Emergency contrast-enhanced abdominal CT revealed splenic artery aneurysm rupture and intraabdominal bleeding, left renal and splenic infarction (Fig.3). Unfortunately, he developed massive bleeding from upper gastrointestinal tract and subsequently cardiopulmonary arrest. Cardiopulmonary resuscitation was unsuccessful and the patient expired. The case was finally diagnosed as infective endocarditis complicated with ruptured infections splenic artery aneurysm and infarction of spleen and left kidney.

## DISCUSSION

Gastrointestinal bleeding caused by rupture of SAAs is very uncommon but catastrophic, and it would be a dangerous pitfall in their diagnosis and management. Herein, we have presented three cases of life-threatening gastrointestinal and intraabdominal bleedings caused by rupture of SAAs in patients with liver cirrhosis or infective endocarditis.

The SAAs are the third commonest among the intraabdominal aneurysms, coming after abdominal aortic and iliac artery aneurysms.^1^ SAAs are a potentially catastrophic clinical entity that can be categorized as true- or pseudo- aneurysms according to the underlying cause. True SAAs are more common and most seen in patients with atherosclerosis, liver cirrhosis complicated with PHTN, or multiple pregnancies, whereas pseudo SAAs are manifested as complications of previous trauma, pancreatitis or infective endocarditis.^2^^,^^3^ Patients with liver cirrhosis accounted for approximate 20% of the total SAAs cases.^2^ The number and size of the SAAs vary greatly between patients. Majority (60%) of SAAs are solitary and <2 cm in diameter, 75% are located in the distal third of splenic artery, followed by 20% in the middle third.^3^^,^^4^

SAAs are difficult to diagnose due to their variable clinical manifestations and insufficient awareness of this rare entity in physicians. Up to 80% of patients with SAA are asymptomatic. The other 20% of patients present with a wide range of nonspecific symptoms including nausea, vomiting, abdominal pain, and bloating.^5^ The expected pulsatile abdominal mass and vascular murmur is only encountered in very few cases because of the SAAs are generally small and located in deep abdomen. Some manifested with hypotension and a sudden collapse as a rupture.^3^^,^^6^ Other complications have been described such as ruptures into a hollow viscus, such as stomach^7^ as in our cases, or the pancreatic duct, and development of splenic arteriovenous fistulas and subsequently sudden onset PHTN. Rupture risk of a symptomatic SAA is reported to be 2% to 3% with mortality approximate 10%~25%. It is increased to 75% to 95% for mother and fetal respectively in pregnant patients.^3^ Noteworthily, as in our record, there is a so-called double rupture phenomenon, which the bleeding may initially remain confined in the lesser omental sac with apparent hemodynamic stabilization. Eventually (usually about 48 hours later), the blood escapes into the free peritoneal cavity, either through the foramen of Winslow or through rupture of the pars flaccida, causing clinical condition deteriorated significantly.

This phenomenon has been reported in 20%~30% of ruptured SAAs.^6^ This double-rupture phenomenon allows valuable time window for diagnosis and surgical intervention. In Case two, the hemoglobin remained relatively stable for 66 hours after the first laparotomy which actually did not define and stop the underlying bleeding cause. After then, he developed hypotension and went into hypovolemic shock. While for Case three, a quickly rebleeding to gastrointestinal tract was catastrophic although hemodynamically he had been primarily stabilized after prior intraabdominal bleeding.

Many SAAs are incidentally found at angiography, laparotomy, or postmortem. Making the diagnosis of SAAs earlier requires a high index of suspicion, especially for patients with PHTN or underlying infective endocarditis, and presented with abdominal pain and symptoms relevant to decrease in intravascular volume such as dizziness or cold sweats. Ultrasonography is not very useful in the diagnosis of SAAs because it is highly operator-dependent and influenced by the intestinal gas and ascites. Arteriography is the gold standard for diagnosis in suspected unruptured aneurysms and is the most valuable tool for diagnosis, localization, assessment of other lesions, and allows percutaneous vascular intervention in the same setting.^8^ Magnetic resonance angiography is highly sensitive and specific; nevertheless it also has its limitations, including long study time, non availability for emergency patients. Multidetector CT, providing excellent image quality and enables volume-rendered image reconstruction, is particularly useful for the diagnosis or an incidental finding of SAAs. In cases of ruptured SAAs present with acute abdominal pain, an emergency CT scan may reveal free fluid in the upper abdomen and may show the aneurysms and leaking of the intravenous contrast if bleeding is in persistent. CT angiography has been reported to have a sensitivity of up to 94.7% and a specificity of 90.0%.^8^ Currently, CT scan with intravenous contrast is the most common diagnostic test due to its availability.^5^^,^^9^

Although SAAs may rupture, not all intact aneurysms need intervention. Most of them remain relatively small and rarely enlarge, become symptomatic, or rupture.^10^ In a consecutively arteriography follow-up of SAAs secondary to liver cirrhosis in six patients it was found that the long diameter of the SAAs only increased by 0.18 mm on average.^11^ Various therapeutic options for SAAs are available which include endovascular management, laparoscopic surgery, and open surgery, although their indications and applications as standard therapy remain controversial. Nevertheless, emergency operative mortality after SAA rupture was nearly 40% compared with zero mortality for elective repair of SAA while patients with PHTN was associated with a higher mortality (56% versus 17%, respectively).^12^ Therefore, recommended indications for early intervention are:^2^^,^^4^ (1) Symptomatic; (2) Greater than 2 cm; (3) Documented enlargement; (4) Women of childbearing age or pregnancy; (5) Portal hypertension; (6) Liver transplantation candidates.

Traditionally, open laparotomy with either aneurysm ligation alone or splenectomy with/without distal pancreatectomy has been the gold standard for the management of SAAs. Preservation of the spleen should be attempted avoiding a lifelong risk of immunologic deficits unless the SAAs are located deep within the splenic hilum, or when associated with other splenic abnormalities, unsalvageable splenic injury, and dissection requiring short gastric vessels sacrifice.^13^ In case of hypovolemic patients due to acute rupture of SAAs, the exclusion by clamping or by ligation of the aneurysm must be the first step during surgery. The further procedure depends on the technical operability, the perfusion situation of the depending parenchyma, and the overall condition of the patient regarding age and comorbidities.

In past years, endovascular therapy of SAAs including coil embolization or stent graft has become increasingly popular.^14^ Endovascular techniques offer a nonsurgical alternative to SAAs management and its success rate varies, depending on the applied technique and the type of aneurysm. Super-selection to the splenic arterial and embolization in splenic artery segments proximal and distal to the aneurysmal neck, so-called sandwich method, is particularly suitable for SAAs in middle third or high risk patients. Though these minimally invasive endoluminal techniques may offer a distinct advantage to conventional repair, overall success rate (75%~92%) is relatively lower compared to open surgery and there is a 20% and 40% rate of early and late failure rates respectively. Moreover, complications like splenic infarction or abscess, stent migration is common.^4^ In Case two, a successful “sandwich” embolization in splenic artery segments failed to stop the bleeding but a conventional open operation saved his life. Covered stent-graft has been steadily gaining popularity for aneurysmal exclusion with a success rate as high as 98%, but the tortuosity of the splenic artery makes this deployment difficult.^15^ Thanks to the improvement of laparoscopic technique, some have successfully performed single resection of SAAs or combined splenecotmy under laparoscopy.^16^

In summary, early diagnosis and intervention for ruptured SAAs are crucial for patient survival. Hence, SAAs must be kept in mind as feasible etiology of life-threatening gastrointestinal bleeding, especially in patients with underlying liver cirrhosis or infective endocarditis.

## References

[1] Grotemeyer D, Duran M, Park EJ (2009). Visceral artery aneurysms—follow-up of 23 patients with 31 aneurysms after surgical or interventional therapy. Langenbecks Arch Surg.

[2] Vlychou M, Kokkinis C, Stathopoulou S (2008). Imaging investigation of a giant splenic artery aneurysm. Angiology.

[3] Pasha SF, Gloviczki P, Stanson AW (2007). Splanchnic artery aneurysms. Mayo Clin Proc.

[4] Ha JF, Sieunarine K (2009). Laparoscopic splenic artery aneurysm resection: review of current trends in management. Surg Laparosc Endosc Percutan Tech.

[5] Roland J, Brody F, Venbrux A (2007). Endovascular management of a splenic artery aneurysm. Surg Laparosc Endosc Percutan Tech.

[6] Zubaidi A (2009). Rupture of multiple splenic artery aneurysms: a common presentation of a rare disease with a review of literature. Saudi J Gastroenterology.

[7] Wierzbicki T, Szmeja J, Borejsza-Wysocki M (2012). Massive bleeeding from upper gastrointestinal tract as a symptom of rupture of splenic artery aneurysm to stomach. Med Sci Monit.

[8] Pasklinsky G, Gasparis AP, Labropoulos N (2009). Endovascular Covered Stenting forvisceral artery pseudoaneurysm rupture. Vasc Endovascular Surg.

[9] Sun C, Liu C, Wang XM (2008). The value of MDCT in diagnosis of splenic artery aneurysms. Eur J Radiol.

[10] Abbas MA, Stone WM, Fowl RJ (2002). Splenic artery aneurysms: two decades experience at Mayo Clinic. Ann Vasc Surg.

[11] Sunagozaka H, Tsuji H, Mizukoshi E (2006). The development and clinical features of splenic aneurysm associated with liver cirrhosis. Liver Int.

[12] Lee PC, Rhee RY, Gordon RY (1999). Management of splenic artery aneurysms: the significance of portal and essential hypertension. J Am Coll Surg.

[13] Kokkalera U, Siddharth B, Ghellai A (2006). Laparoscopic management of splenic artery aneurysms. J Laparoendosc Adv Surg Tech.

[14] Madoff DC, Denys A, Wallace M (2005). Splenic arterial interventions: anatomy, indications, technical considerations, and potential complications. Radiographics.

[15] Briard RJ, Lee J, Doyle T (2009). Endovascular repair of a portal hypertension-related splenic artery aneurysm using a self-expanding stent graft. Cardiovasc Intervent Radiol.

[16] Obuchi T, Sasaki A, Nakajima J (2009). Laparoscopic surgery for splenic artery aneurysm. Surg Laparosc Endosc Percutan Tech.

